# Hypercholesterolemia Interferes with Induction of miR-125b-1-3p in Preconditioned Hearts

**DOI:** 10.3390/ijms21113744

**Published:** 2020-05-26

**Authors:** Márton R. Szabó, Renáta Gáspár, Márton Pipicz, Nóra Zsindely, Petra Diószegi, Márta Sárközy, László Bodai, Tamás Csont

**Affiliations:** 1 Metabolic Diseases and Cell Signaling (MEDICS) Research Group, Department of Biochemistry, Faculty of Medicine, University of Szeged, Dóm tér 9., H-6720 Szeged, Hungary; szabo.marton@med.u-szeged.hu (M.R.S.); gaspar.renata@med.u-szeged.hu (R.G.); pipicz.marton@med.u-szeged.hu (M.P.); dioszegi.petra@med.u-szeged.hu (P.D.); sarkozy.marta@med.u-szeged.hu (M.S.); 2 Interdisciplinary Centre of Excellence, University of Szeged, Dugonics tér 13., H-6720 Szeged, Hungary; 3 Department of Microbiology, Faculty of Science and Informatics, University of Szeged, Közép fasor 52., H-6726 Szeged, Hungary; zsindizsn@yahoo.com; 4 Department of Biochemistry and Molecular Biology, Faculty of Science and Informatics, University of Szeged, Közép fasor 52., H-6726 Szeged, Hungary; bodai@bio.u-szeged.hu

**Keywords:** hypercholesterolaemia, cardioprotection, miR-125b, miR-125b*, miRNA, protectomiR, risk factor, comorbidity, RISK, SAFE

## Abstract

Ischemic preconditioning (IPre) reduces ischemia/reperfusion (I/R) injury in the heart. The non-coding microRNA miR-125b-1-3p has been demonstrated to play a role in the mechanism of IPre. Hypercholesterolemia is known to attenuate the cardioprotective effect of preconditioning; nevertheless, the exact underlying mechanisms are not clear. Here we investigated, whether hypercholesterolemia influences the induction of miR-125b-1-3p by IPre. Male Wistar rats were fed with a rodent chow supplemented with 2% cholesterol and 0.25% sodium-cholate hydrate for 8 weeks to induce high blood cholesterol levels. The hearts of normo- and hypercholesterolemic animals were then isolated and perfused according to Langendorff, and were subjected to 35 min global ischemia and 120 min reperfusion with or without IPre (3 × 5 min I/R cycles applied before index ischemia). IPre significantly reduced infarct size in the hearts of normocholesterolemic rats; however, IPre was ineffective in the hearts of hypercholesterolemic animals. Similarly, miR-125b-1-3p was upregulated by IPre in hearts of normocholesterolemic rats, while in the hearts of hypercholesterolemic animals IPre failed to increase miR-125b-1-3p significantly. Phosphorylation of cardiac Akt, ERK, and STAT3 was not significantly different in any of the groups at the end of reperfusion. Based on these results we propose here that hypercholesterolemia attenuates the upregulation of miR-125b-1-3p by IPre, which seems to be associated with the loss of cardioprotection.

## 1. Introduction

Myocardial infarction, characterized by restriction of blood flow to the myocardium, is a major cause of death worldwide [1,2]. Nevertheless, the heart is able to adapt remarkably to withstand the detrimental effects of ischemic injury. One of the most powerful strategies to trigger endogenous cardioprotective mechanisms in the myocardium is ischemic preconditioning (IPre), i.e., when short, repetitive cycles of ischemia/reperfusion (I/R) are applied before the sustained lethal ischemia [3]. The molecular mechanism of IPre is complex and still not entirely clear. Trigger molecules (e.g., adenosine, opioids, bradykinin, and nitric oxide) induced by preconditioning has been shown to activate G-protein-coupled receptors and cardioprotective protein kinases (e.g., Akt, Erk, STAT3, and PKG), thereby leading to cell survival [4,5]. Besides activation of several signaling pathways, changes in protein-coding gene expression of the heart in response to preconditioning have been demonstrated, too. Moreover, non-coding microRNAs (miRNA) have emerged as regulators of preconditioning [6]. Indeed, we have previously shown that preconditioning alters the expression of several miRNAs in the heart in settings of ischemia/reperfusion [7]. We have identified miR-125b*, more specifically miR-125b-1-3p, as a cardioprotective miRNA, since preconditioning enhanced its expression and cardiac cells transfected with miR-125b* mimics showed significant survival rate during simulated ischemia/reperfusion injury [7].

Interestingly, metabolic diseases like diabetes or hyperlipidemia may interfere with the efficacy of IPre [8]. Hypercholesterolemia is a well-known risk factor for myocardial infarction due to its promoting effect on coronary atherosclerosis [2]. Nevertheless, independently from its proatherogenic effect, a high blood cholesterol level exerts a direct adverse effect on the myocardium leading to cardiac dysfunction, altered tolerance to I/R injury, and disturbed stress adaptation [8,9]. High cholesterol level is thought to induce oxidative/nitrosative stress in the myocardium [9,10], thereby leading to dysregulation of cardioprotective signaling pathways (e.g., decreases in nitric oxide bioavailability, and in activation of cardioprotective protein kinases). In addition, hypercholesterolemia was shown to alter cardiac gene expression of mRNAs as well as miRNAs. We have demonstrated for instance, that downregulation of cardiac miR-25 in hypercholesterolemic rats mediates oxidative/nitrative stress in the heart [11]. Nevertheless, the exact underlying mechanisms responsible for the attenuated cardioprotective effect of preconditioning in hypercholesterolemia are unclear; therefore we investigated whether hypercholesterolemia influences miR-125b-1-3p upregulation induced by preconditioning.

## 2. Results

### 2.1. Verification of Hypercholesterolemia

**Figure 1:**
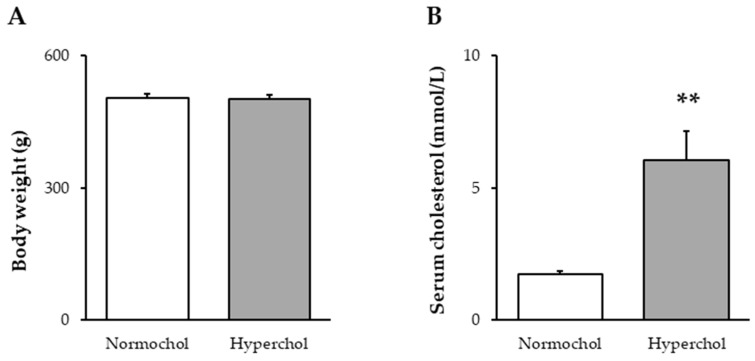
Body weight (**A**) and serum total cholesterol levels (**B**) at the end of 8 weeks of cholesterol diet. Data are expressed as mean ± SEM; *n* = 14–16. ** *p* < 0.01 vs. Normochol. Normochol and Hyperchol refer to normo- and hypercholesterolemia, respectively.

At the end of the 8-week diet period, the body weight of animals receiving cholesterol-enriched diet was not different from the weight of control animals fed a standard diet (Figure 1A). In order to verify the development of hypercholesterolemia in our model, total blood cholesterol level was measured. Total cholesterol was significantly higher in cholesterol-fed rats when compared to control animals fed with standard chow (Figure 1B).

### 2.2. Hypercholesterolemia Attenuates the Infarct Size-Limiting Effect of Ischemic Preconditioning

**Figure 2:**
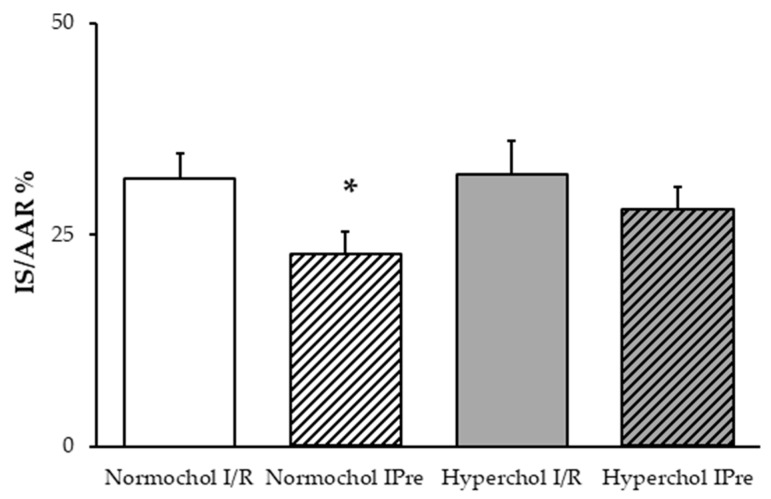
Infarct size values at the end of ex vivo heart perfusion. Hearts isolated from normo- and hypercholesterolemic rats were subjected to 35 min global ischemia and 120 min reperfusion (ischemia/reperfusion (I/R)) with or without ischemic preconditioning (3 × 5 min cycles of I/R applied before index ischemia; IPre). IS/AAR = infarct size/area at risk %. Data are expressed as mean ± SEM; *n* = 8. * *p* < 0.05 vs. corresponding I/R group. Normochol and Hyperchol refer to normo- and hypercholesterolemia, respectively.

To assess the cardioprotective effect of IPre, infarct size was measured in hearts undergoing I/R. In the hearts of normocholesterolemic rats, IPre significantly decreased infarct size compared to the I/R control group (Figure 2). However, IPre failed to significantly attenuate infarct size in the hearts of hypercholesterolemic animals (Figure 2). 

### 2.3. Upregulation of miR-125b-1-3p Induced by Preconditioning is Lost in Settings of Hypercholesterolemia

**Figure 3:**
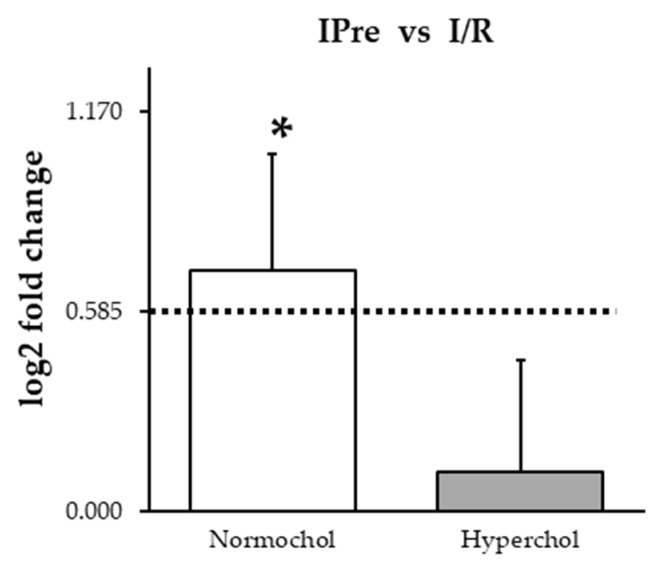
miR-125b-1-3p expression changes induced by ischemic preconditioning (IPre) in hearts of normocholesterolemic and hypercholesterolemic rats. Values are log2 expression changes ± SEM calculated with Deseq2. * *p* < 0.05 and log2 fold change is greater than 0.585 vs. corresponding ischemia/reperfusion (I/R) control group. Normochol and Hyperchol refer to normo- and hypercholesterolemia, respectively.

In order to assess if miR-125b-1-3p correlates with cardioprotection, miRNA expression was determined in hearts subjected to I/R with or without IPre in both normo- and hypercholesterolemic groups. At the end of reperfusion, IPre significantly upregulated miR-125b-1-3p in normocholesterolemic hearts compared to I/R controls (Figure 3). In contrast, IPre failed to increase significantly miR-125b-1-3p level in hearts of hypercholesterolemic animals (Figure 3).

### 2.4. Ischemic Preconditioning Failed to Affect the RISK and SAFE Pathways at the End of Reperfusion

**Figure 4:**
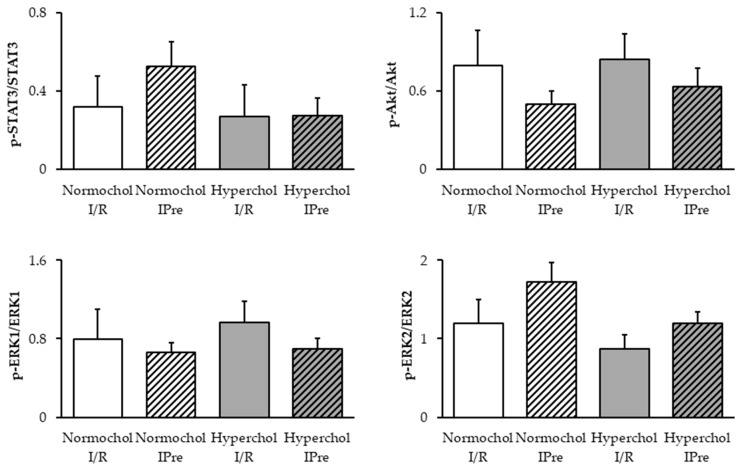
Delayed phosphorylation of STAT3, Akt, and ERK1/2 proteins assessed by Western blots. Ventricular samples were harvested at the end of reperfusion from normo- and hypercholesterolemic hearts subjected to ischemia/reperfusion (I/R) with or without ischemic preconditioning (IPre). Data are expressed as mean ± SEM; *n* = 5, Two-way ANOVA. Normochol and Hyperchol refer to normo- and hypercholesterolemia, respectively.

To elucidate the possible downstream mechanism of IPre in normo- and hypercholesterolemic conditons, Reperfusion Injury Salvage Kinases (RISK) and Survivor Activating Factor Enhancement (SAFE) pathways were investigated in ventricular samples obtained at the end of reperfusion. Although, a slight decrease of Akt phosphorylation and a slight increase in ERK2 and STAT3 phosphorylation may be seen in preconditioned normocholesterolemic hearts compared to I/R controls, the phosphorylation of Akt, ERK1/2, and STAT3 were not affected significantly by any of the interventions (Figure 4). In hypercholesterolemic groups phosphorylation of Akt, ERK1/2, and STAT3 were not affected by IPre. 

## 3. Discussion

In the present study, we have shown an association of the attenuated cardioprotective effect of IPre in hypercholesterolemia with diminished miR-125b-1-3p induction. This is the first demonstration that diet-induced hypercholesterolemia blunts the cardiac overexpression of miR-125b-1-3p triggered by IPre. Together with previous findings, our present results suggest that miR-125b-1-3p may be an important activator of IPre-induced cardioprotection and its decreased expression level seems to interfere with the infarct size limiting effect of IPre in hypercholesterolemia. 

Similarly to literature data [5], in our isolated perfused heart model, the application of IPre in normocholesterolemic hearts decreased infarct size compared to the I/R group. IPre is one of the most powerful endogenous cardioprotective approaches as it markedly enhances the ability of the heart to withstand a subsequent ischemic injury. The early phase of IPre (i.e., classic preconditioning or first window of protection) manifested within minutes after preconditioning stimulus, was first described by Murry and colleagues [3]. The protective effect of IPre against myocardial infarction was confirmed using numerous species in several experimental models [4]. Although a number of signaling pathways have been implicated in IPre, the exact mechanism is still not entirely clear.

Non-protein-coding miRNAs have been proposed to play a role in IPre [4,5,7]. MiR-125b-1-3p (previously named miR-125b*) is the passenger antisense strand of miR-125b-1 stem-loop precursor. Antisense miRNAs are considered to be degraded during miRNA maturation. However, recent data suggest that passenger strands also have biological functions [12,13]. So far, only a few studies have revealed that miR-125b-1 protects the heart against I/R injury. In the present study, we investigated the expression levels of miR-125b-1-3p in ex vivo perfused hearts, and we showed upregulation of miR-125b-1-3p by IPre in normocholesterolemic hearts, suggesting that miR-125b-1-3p may play a role in IPre-induced cardioprotection. This finding is in accordance with our previous studies, when we identified miR-125b-1-3p as a protectomiR since both IPre and postconditioning induced miR-125b-1-3p expression in the setting of I/R [7]. Moreover, in the same study, cardiomyocytes transfected with miR-125b-1-3p showed enhanced cell viability following simulated I/R injury. In contrast, Li and colleagues found that decreased level of miR-125b-1-3p is associated with rutin-induced cardioprotection in HL-1 cells [14]. These discrepancies may arise due to substantial differences in the applied models since the molecular mechanism of I/R may differ from the mechanisms of drug–induced cardiotoxicity. Interestingly, literature data support the cardioprotective effect of the predominantly expressed sense strand of mature miR-125b-1 as well. Myocardial infarction was lower in precursor miR-125b-1 (encoding both 125b-1-3p and -5p) overexpressing transgenic mice, possibly due to inhibition of apoptotic signaling [15]. In a separate study, pharmacological induction of miR-125b-5p conferred cardioprotection against ischemia/reperfusion by suppressing Bak1 and Kfl13 protein expressions [16]. Furthermore, pretreatment with miR-125b-5p containing exosomes derived from mesenchymal stem cells protected the murine heart from myocardial infarction and cultured cardiomyocytes against simulated I/R injury [17,18]. Nevertheless, the expression of miR-125b-5p seems to have adverse effects in the failing heart through inducing cardiac fibrosis [19,20,21]. 

In the present study, we have found that hypercholesterolemia abolished the infarct size limiting effect of IPre. This is in line with previous reports from our group and others, demonstrating that hypercholesterolemia interferes with cardioprotection [22,23,24,25,26]. However, here we found that in contrast to findings obtained in normocholesterolemia, the application of IPre in hypercholesterolemia failed to upregulate miR-125b-1-3p expression as compared to I/R. We have previously demonstrated that diet-induced hypercholesterolemia alters cardiac miRNA expression profile, and as a consequence, downregulation of miR-25 mediates oxidative/nitrosative stress in the myocardium [11]. Although several studies revealed relationships between IPre and cardiac miRNA levels in normocholesterolemic subjects, this is the first demonstration that hypercholesterolemia influences miRNA expression changes induced by IPre. Our study suggests that the upregulation of miR-125b-1-3p in response to IPre may be an important element in the cardioprotective mechanism; however, further studies need to confirm a direct causative relationship. 

In our current study, we also looked at possible downstream signaling mechanism of IPre as an attempt to relate the observed alterations in miR-125b-1-3p expression with known cardioprotective mechanisms. Therefore, we have assessed phosphorylation rates of Akt, ERK1/2, and STAT3 at the end of reperfusion, as both infarct size and miR analysis was performed at that timepoint in the current study. IPre in normocholesterolemic hearts failed to increase significantly the delayed phosphorylation of Akt, ERK1/2, and STAT3 proteins, respectively. This seems to be against the general view that these kinases contribute to the protective effect of IPre. The discrepancies may be explained by the experimental protocol that we used. We have determined kinase phosphorylations 2 h after the onset of reperfusion, while the phosphorylation status of IPre-related kinases is mostly investigated at the beginning of reperfusion. Nevertheless, some studies have already assessed the late activation of cardiac RISK and SAFE pathways after 30, 120, or 180 min of reperfusion in response to ischemic conditionings [27,28,29]. Our present findings do not exclude the possible activation of the RISK and/or SAFE pathways by IPre in earlier phases of the protocol we used. However, at the late phase, when miR-125b-1-3p induction was evident, the activation of RISK and SAFE pathways does not seem to play a crucial role in the cardioprotective mechanism of IPre. This may be a valuable conclusion as some other microRNAs has been linked to STAT3 modulation, e.g., miR-874 inhibition targeting STAT3 has been shown to protect the heart against I/R injury by attenuating cardiomyocyte apoptosis in a mouse model [30]. Based on our results, one may speculate that upregulation of miR-125b-1-3p is rather a consequence of the activation of RISK and SAFE pathways; however, further research is needed to prove this hypothesis. We have found in the current study that IPre failed to affect significantly the delayed phosphorylation of Akt and ERK1/2 proteins in hypercholesterolemic hearts. Interestingly, Akt may stimulate nitric oxide production via activation of endothelial nitric oxide synthase (eNOS) in response to preconditioning stimuli [31,32] and a decreased cardiac nitric oxide content was suggested to correlate with impaired cardioprotection due to IPre in cholesterol-fed rats [9,22,33]. Based on our present results, it is unlikely that miR-125b-1-3p would regulate Akt-eNOS-nitric oxide-PKG signaling in our model, however, whether nitric oxide is able to affect cardiac miR-125b-1-3p expression in normocholesterolemia needs to be assessed in the future. Our current results also show that hypercholesterolemia does not affect STAT3 phosphorylation after I/R with or without IPre, thereby providing a deeper insights into the rather unclear effects of metabolic disorders on cardiac STAT3 signaling [34].

Similarly to all experimental investigations, our study is not without limitations. We have analyzed microRNA expression and the RISK and SAFE pathways at a single time-point, i.e., at 2 h of reperfusion. This may limit proper interpretation of early molecular changes in the course of cardioprotection, therefore assaying miR-125b-1-3p, triggers for miR-125-b-1-3p induction and survival kinases at earlier time-points in future studies may have some added value. Although it is clinically less relevant, a common feature of studies performed on I/R—including ours—is that the ischemic tissue may contain both viable and non-viable cells, and the lack of separation of these cells for biochemical analysis may potentially affect overall molecular markers of the tissue. This is especially problematic when regional ischemia is used, because it is almost impossible to separate the non-ischemic, ischemic-viable, and ischemic-non-viable cells. Therefore, in the present study we applied global ischemia, where all cells were exposed to the same stress (i.e., ischemia). Despite these limitations our study provides valuable data regarding the effect of experimental hypercholesterolemia on some aspects of the potential molecular mechanism of ischemic preconditioning.

We conclude that miR-125b-1-3p upregulation is an adaptive response to prevent cellular damage induced by I/R, however, the exact role and molecular targets of miR-125b-1-3p should be further analyzed in future studies. This is the first demonstration that hypercholesterolemia attenuates IPre-induced miR-125b-1-3p upregulation, which is likely associated with the loss of cardioprotection. These results may suggest that modulation of cardiac miR-125b-1-3p could be a feasible target for cardioprotection even in cases when risk factors and comorbidities are present; however, this hypothesis remained to be confirmed in future experimental studies.

## 4. Materials and Methods 

### 4.1. Animals

A total of 56 adult male Wistar rats were used in this study. All experiments conformed to the Guide for the Care and Use of Laboratory Animals published by the US National Institutes of Health (NIH publication No. 85-23, revised 1996) and was approved by the Animal Research Ethics Committee of Csongrád County (approval code: XV.1181/2013) and the local animal ethics committee of the University of Szeged.

### 4.2. Experimental Setup

**Figure 5:**
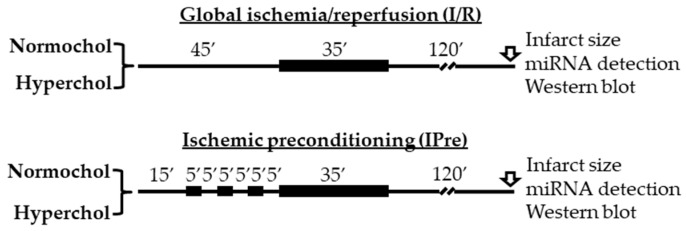
Experimental protocols for ex vivo ischemia/reperfusion (IR) and IR with ischemic preconditioning (IPre). At the end of reperfusion infarct size, miRNA expression analyses and Western blotting measurements were performed from the left ventricles. Normochol and Hyperchol refer to normo- and hypercholesterolemia, respectively.

Male Wistar rats (250–300 g) were fed for 8 weeks with a laboratory chow enriched with 2% (*w/w*) cholesterol and 0.25% sodium-cholate hydrate (*w/w*). The control animals were fed with standard rodent chow. At the end of the diet period rats were anesthetized with intraperitoneal injection of sodium pentobarbital (50 mg/kg; Produlab Pharma b.v., Raamsdonksveer, The Netherlands), blood samples were collected from the thoracic aorta and hearts were isolated and placed into ice-cold Krebs–Henseleit buffer. After cannulation of the aorta, isolated hearts were perfused with an oxygenated Krebs–Henseleit buffer at 37 °C in a retrograde manner according to Langendorff as described previously [35,36,37]. Then hearts from both feeding groups were divided into global ischemia/reperfusion (I/R) or ischemic preconditioning (IPre) groups (Figure 5). The time-matched I/R control group hearts were equilibrated for 45 min before 35 min global ischemia and 120 min reperfusion. In the IPre group after 15 min equilibration time IPre was induced by 3 intermittent cycles of 5 min no-flow ischemia, separated by 5 min aerobic perfusion before the onset of global ischemia. At 120 min after the onset of reperfusion, either infarct size was determined (*n* = 8 in each group) or left ventricles were snap-frozen in liquid nitrogen and stored at −80 °C for miRNA analysis and Western blot experiments (*n* = 6 in each group). Global ischemia was used in this study in order to apply the same degree of stress (i.e., ischemia) for the entire myocardium.

### 4.3. Serum Total Cholesterol Measurement

Serum was separated from the freshly collected blood samples and used to determine total cholesterol concentrations using a colorimetric cholesterol detecting kit (Diagnosticum, Budapest, Hungary) and a microplate reader (BMG Labtech, Ortenberg, Germany) as described previously [10,11].

### 4.4. Infarct Size Determination

In a separate set of experiments, hearts were isolated and perfused as described above. After the end of reperfusion, atria were removed, and the total ventricles were used to determine the infarcted area as described previously [38,39]. Briefly, frozen ventricles were cut to 7–8 equal slices and placed into triphenyl-tetrazoliumchloride solution (Sigma-Aldrich, Saint Louis, MO, USA) for 10 min at 37 °C followed by a 10 min formaldehyde fixation and phosphate buffer washing steps. As a result, survived area were red-stained while the necrotic area become pale. Digitalized images from the stained heart slices were evaluated with planimetry method and the amount of myocardial necrosis was expressed as infarct size/area at risk %.

### 4.5. Measurement of Cardiac miR-125b-1-3p Level

Six heart sample from each group were used for miRNA-sequencing. Total RNA was isolated from left ventricles with miRNeasy Mini kit (Qiagen, Hilden, Germany). Total RNA samples were quality checked and quantified by capillary gel electrophoresis in an Agilent Bioanalyzer 2100 instrument using Agilent 6000 RNA Nano Kit (Agilent Technologies, Santa Clara, CA, USA). Then 1000 ng total RNA samples were used to prepare sequencing libraries using NEBNext Multiplex Small RNA Library Prep Set for Illumina (New England Biolabs, Iswich, MA, USA) following the recommendations of the manufacturer. Sequencing libraries were size selected with AMPure XP beads (Beckman Coulter, Pasadena, CA, USA) then validated and quantitated in an Agilent 2100 Bioalayzer using Agilent DNA 1000 kit. Validated library pools were denatured and loaded in MiSeq Reagent Kit V3-150 (New England Biolabs, Iswich, MA, USA) and sequenced with an Illumina MiSeq (Illumina Inc, San Diego, CA, USA) instrument generating 36 nucleotides long single-end reads.

### 4.6. Bioinformatic Analysis

Sequencing reads were quality checked with FastQC (Babraham Bioinformatics, UK) and adapter trimmed using Cutadapt ver. 1.8.1 [40]. Trimmed oligos were analyzed with mirdeep2 [41]. Reads were aligned to the rat reference genome using the mapper module than sequenced reads were mapped to predefined miRNA precursors, and the expression of the corresponding miRNAs were determined using the quantifier module. Differential expression analysis were performed with Deseq2 [42]. 

### 4.7. Western Blotting

Frozen left ventricular samples were homogenized in Radio Immunoprecipitation Assay buffer (Cell Signaling Technology, Danvers, MA, USA) supplemented with protease inhibitor cocktail, phenylmethane sulfonyl fluoride and sodium fluoride (Sigma-Aldrich, Saint Louis, MO, USA) as described previously [37,43,44,45]. Homogenates were centrifuged, and protein concentrations of the supernatants were determined using BCA Protein Assay Kit (Pierce, Rockford, IL, USA). Twenty-five micrograms of reduced and denatured protein was loaded in 10% polyacrylamide gel, and SDS gel electrophoresis was performed. Separated proteins were transferred to 0.22 μM pore size nitrocellulose membranes. After checking, the transfer efficiency with Ponceau-stained membranes were blocked for 1 h in 5% (*w/v*) bovine serum albumin (Sigma-Aldrich, Saint Louis, MO, USA) at room temperature. Blocked membranes were incubated with the following primary antibodies in the concentrations of 1:1000 phospho-Akt (Ser473, #4060), Akt (#9272), phospho-ERK1/2 (Thr202/Tyr204, #9101), ERK1/2 (#9102), phospho-STAT3 (Tyr705, #9145), STAT3 (#4904 and in 1:5000 concentration against GAPDH (#2118) at 4 °C overnight (Cell Signaling Technology, Danvers, MA, USA). After incubation with horseradish peroxidase (HRP)-conjugated goat anti-rabbit secondary antibody (Dako, Glostrup, Denmark) membranes were developed with an enhanced chemiluminescence kit. After development of phosphorylated signals of Akt, Erk1/2, and STAT3, respectively, the stripped membranes were reassessed for the total amount of proteins. The developed signals were evaluated by Quantity One Software (Bio-Rad, Hercules, CA, USA).

### 4.8. Statistical Analysis

Values are expressed as mean ± SEM. Student’s t-test was used to evaluate the effect of cholesterol-enriched diet in body weight and serum cholesterol level, while two-way analysis of variance (ANOVA) was used to evaluate infarct size values and Western blotting results. Wald test was performed in differential miRNA expression analysis. MiRNA expression ratio with *p*-value <0.05 and >1.5 fold change (0.585 log2 fold change) are considered as significant expression difference. 

## Abbreviations

I/R	Ischemia/Reperfusion
IPre	Ischemic Preconditioning
miRNA PKG RISK	microRNA Protein kinase G Reperfusion Injury Salvage Kinases
SAFE	Survivor Activating Factor Enhancement pathway
